# All-optical processors by 3D printable photochromic materials

**DOI:** 10.1038/s41377-025-01974-z

**Published:** 2025-10-22

**Authors:** Francesca D’Elia, Lorenzo Lavista, Sibilla Orsini, Andrea Camposeo, Dario Pisignano

**Affiliations:** 1 NEST, Scuola Normale Superiore, Pisa, 56127 Italy; 2 Dipartimento di Fisica, Università di Pisa, Pisa, 56127 Italy; 3 NEST, Istituto Nanoscienze-CNR and Scuola Normale Superiore, Pisa, 56127 Italy

**Keywords:** Polymers, Lithography

## Abstract

Developing new responsive materials whose physico-chemical properties can be controlled and tailored by external stimuli is fundamental for many modern technologies. In this framework, 3D-printable photochromic materials and systems for all-optical data processing might enable remote addressing, by optical control of their response with high spatiotemporal accuracy, thus supporting the development of new computing and sensing platforms with multidimensional fashion. Here, we introduce 3D-printable photochromic materials based on either a spiropyran molecular system or a diarylethene derivative shaped by digital light processing. Dynamically controlling transmitted light by the intensity and sequence of incoming signals, these materials exhibit robust photoswitching cycles, long-term optically-textured information storage, and are used in 3D printed devices capable of all-optical arithmetic and logic processing. These compounds and devices open a route to new 3D all-organic all-optical computing platforms, and to new schemes and architectures for advanced microscopy, sensing, and physical intelligence.

## Abstract (web summary)

3D-printable photochromic materials are introduced and applications in all-optical data-processing shown. 3D-printed photochromic processors show robust photoswitching cycles, long-term optically-textured information storage, and capability to perform arithmetic and logic processing.

## Graphical abstract

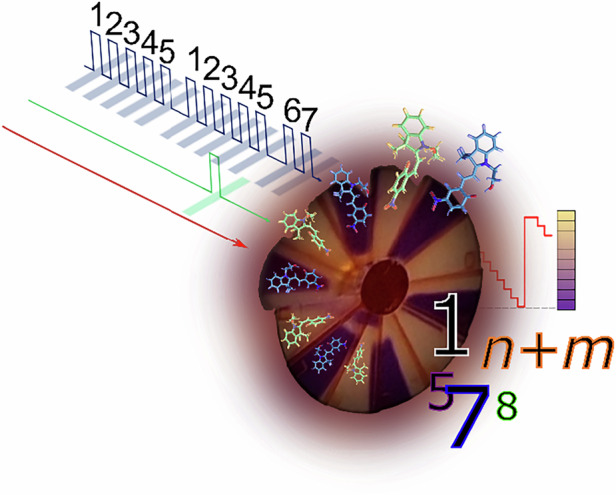

## Introduction

Materials with properties controlled by external stimuli are highly desired for many and diverse applications^1–5^, and various responsive molecules or supramolecular systems^6–8^ can be used to this aim. Importantly, addressing the properties of such systems once in practical, macroscopic components also requires that precisely-designed shapes and geometries are achieved in compound materials. These patterns are crucial, for instance, to create devices that are reconfigurable^9–11^ and ultimately adaptive, and that are able to optically perform logic operations and dense data storage^12,13^. In this framework, photoresponsive systems, such as photochromic molecules that feature ultraviolet (UV) light-triggered transition from a colorless to a colored state, are particularly appealing to embed in printable materials^14^. The photochromic behavior might involve structural molecular rearrangements that modify the π-conjugation extent, featuring reversible isomerization, and back-conversion occurring either by thermal relaxation or upon illuminating the material with visible light. These properties have been exploited in a variety of solution or polymer-dispersed systems to obtain switches^15^, spectral selectors^16^, data storage components^17^, logic gates^18–20^, artificial synapses^21^, and anticounterfeiting elements^22^. Photochromic molecules in 3D-printable materials have been used to control photopolymerization processes^23–25^, and to build smart actuators^26^, 3D memories^27^, and responsive micro-objects^28^. Developing 3D-printed photochromic systems for all-optical data processing would be critically important for many modern technologies, enabling remote addressing with high spatiotemporal accuracy, as well as new computing and sensing platforms. However, these materials are still largely unexplored.

Here, we report on 3D-printable photochromic materials that allow us to control the attenuation of light passing through them, both spatially and temporally, which enables all-optical arithmetic and logic processing in a 3D fashion. Two systems are introduced, based on a spiropyran and a diarylethene derivative, respectively. Molecules are incorporated in a photocurable pre-polymer and printed by digital light processing (DLP), then featuring robust photoswitching cycling, and optically-textured information storage over months. Being precisely controlled by the intensity and sequence of incoming light signals, the response properties of these compounds and devices open a route to new 3D architectures where computing, information storage and control of light propagation are synergistically integrated.

## Results

**Fig. 1:**
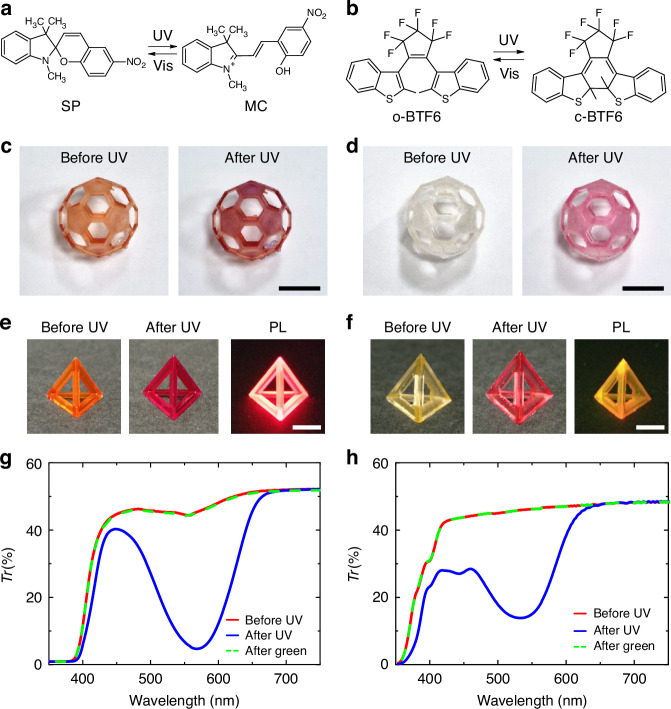
3D printable photochromic materials. Molecular structures of the photochromic molecules SP, MC (**a**), and o-BTF6, c-BTF6 (**b**). Photographs of 3D-printed photochromic objects with SP/MC (**c**) and BTF6 (**d**) before (left images) and after (right images) UV exposure. Objects are made of BEDMA, with *χ*_TPO_ = 0.5%, *χ*_SP_ = 1% and *χ*_BTF6_ = 1%. Scale bars: 5 mm. Photographs of 3D-printed pyramids with SP/MC (**e**) and BTF6 (**f**) upon ambient illumination before (left images) and after (middle images) the exposure with UV light. The pyramids are made of BEDMA, with *χ*_TPO_ = 0.5%, *χ*_SP_ = 3.5%, and *χ*_BTF6_ = 3%. Scale bars: 1 cm. The images on the right sides show the PL of the printed pyramids upon UV excitation (365 nm). Transmittance (*Tr*) spectra of 3D-printed slabs made of BEDMA doped with SP/MC (**g**) and BTF6 (**h**), and with same composition as in (**e**) and (**f**), respectively. *Tr* is measured in pristine samples (red continuous lines), after UV exposure (blue continuous lines), and after consecutive UV and green exposures (green dashed lines)

The 3D-printable photochromic materials are made of the oligomer bisphenol A ethoxylate dimethacrylate (BEDMA) and the photoinitiator diphenyl(2,4,6-trimethylbenzoyl) phosphine oxide (TPO). BEDMA is selected as a matrix that can offer both optical transparency in the visible spectral range (Fig. S1 of the Supplementary Information -SI-) and high structural stability upon UV photopolymerization. The TPO/BEDMA pre-polymer is doped with either 1′,3′-dihydro-1′,3′,3′-trimethyl-6-nitrospiro[2H-1-benzopyran-2,2′-(2H)-indole] (SP, Fig. 1a) or 1,2-bis(2-methyl-1-benzothiophene-3-yl) perfluorocyclopentene (BTF6, Fig. 1b). The colorless SP and o-BTF6 forms are converted to colored merocyanine (MC) and to c-BTF6, respectively, by means of UV exposure. Several compounds with varying weight ratios of TPO (*χ*_TPO_), SP (*χ*_SP_) and BTF6 (*χ*_BTF6_) compared to the BEDMA matrix are investigated in order to obtain uniform printed objects (see Materials and Methods Section for details), as shown in Fig. 1c-f. When illuminated with UV light, the 3D structures show the characteristic colorless-to-colored transition of the two photochromic molecules. Control experiments, performed with 3D printed BEDMA without photochromic molecules, do not shown any color transition upon UV and green irradiation (insets of Fig. S1). Samples with different degree of crosslinking show quite similar color conversion behavior (Fig. S2). Further, both MC and c-BTF6 are also fluorescent (Fig. 1e,f and Fig. S3). The photoluminescence (PL) of MC (c-BTF6) in printed objects is peaked at about 654 nm (617 nm), with full width at half maximum of 62 nm (75 nm), well-retaining the typical spectral signatures of the used molecules^29,30^. Figure 1g and 1h show the UV-visible spectra of the light transmitted through printed samples with SP and BTF6 molecules, respectively, measured before and after UV exposure. The UV-triggered isomerization leads to the appearance of a band in the visible, consistent with MC and c-BTF6 absorption at ∼565 nm (Fig. 1g) and ∼535 nm (Fig. 1h), respectively.

Then, exposure with green light excellently reverts back the two systems, suppressing the absorption bands characteristic of MC and c-BTF6 and allowing the pristine optical spectra to be fully recovered (dashed curves in Fig. 1g and h, and Figs. S4b,d and S5b,d).

The transmission spectra also allow us to assess the temporal evolution of photoisomerization processes in printed samples (Figs. S4 and S5). The SP→MC conversion reaches a photostationary state upon UV exposure by about 30 s (Fig. S4a,c). Back-conversion by green light leads transmittance to its initial values after about 70 s (Fig. S4b,d). A similar behavior is observed for BTF6 (Fig. S5).

**Fig. 2:**
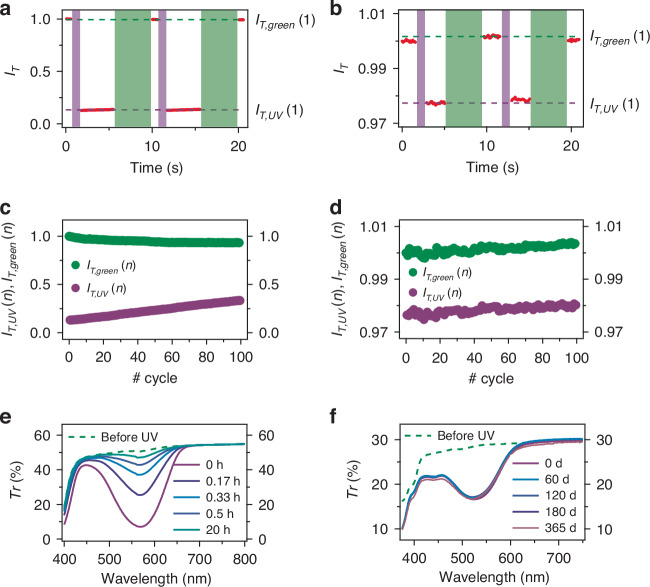
. **Stability properties and back-conversion**. Intensity (*I*_*T*_) of a probe light beam at 617 nm, transmitted through SP/MC (**a**) and BTF6 printed samples (**b**), upon cyclic exposure with UV and green light. *I*_*T*_ values (red data) are normalized to the transmission intensities at the initial instant. The sequences of UV and green exposure intervals are shown as violet and green shadowed areas in the figures. The average values of *I*_*T*_ for samples after the first UV [*I*_*T,UV*_(1)] and the first green [*I*_*T,green*_(1)] exposure are highlighted by dashed horizontal violet and green lines. *I*_*T,UV*_(*n*) and *I*_*T,green*_(*n*) over 10^2^ consecutive UV/green exposure cycles for SP/MC (**c**) and for BTF6 printed samples (**d**). **e** Transmission spectra of pristine SP/MC printed samples (dashed line), and after UV irradiation and storage in dark for different time intervals, *t*_*dark*_ (continuous lines). From bottom to top, *t*_*dark*_ = 0, 0.17, 0.33, 0.5, 5, 20 hours. **f** Transmission spectra of pristine BTF6 printed samples (dashed line), and after UV irradiation and storage in dark for *t*_*dark*_ = 0, 60, 120, 180 and 365 days (continuous lines)

Figure 2a,b displays the variation of the intensity, *I*_*T*_, of a probe light beam (617 nm), passing through SP/MC and BTF6 printed samples, respectively, following consecutive UV and green exposure (set-up in Fig. S6a). The stability properties of the printed photochromic materials are shown by the dependence of *I*_*T*_ on the number (*n*) of performed exposure cycles (Fig. 2c,d). For SP/MC samples after 10^2^ cycles, the differential transmission, *I*_*T,green*_(*n*) - *I*_*T,UV*_(*n*), between the probe intensities transmitted after a green and a UV exposure step, is still about 70% of the initial value. Such fatigue behavior can be attributed to photo-oxidation effects^31^, photoisomerization towards poorly back-converting isomers^32^, or aggregate formation^33^. Bleaching and aggregation processes could decrease the available photoisomerizable molecules, thus decreasing MC-associated optical absorption (i.e., increasing *I*_*T,UV*_). In addition, aggregates might also contribute to a decrease of the sample transparency by light-scattering (thus contributing to *I*_*T,green*_ decrease). Importantly, the fatigue behavior here observed is significantly improved compared to SP/MC systems in other polymer matrices^32^. An even better stability is measured for BTF6 printed samples, for which the differential transmission keeps at 85% of the initial value after 10^2^ UV/green exposure cycles. The transmission spectra of SP/MC and BTF6 printed samples, in dark and at ambient temperature upon thermal relaxation, are displayed in Figs. 2e and 2f, respectively. While MC is almost back-reverted in SP in about 1 hour (Fig. S6b), the spectra measured with BTF6 samples are almost unchanged after twelve months, evidencing the excellent stability^34^ of this 3D-printed photochromic system and making it very well-suited for encoding and data storage applications.

**Fig. 3:**
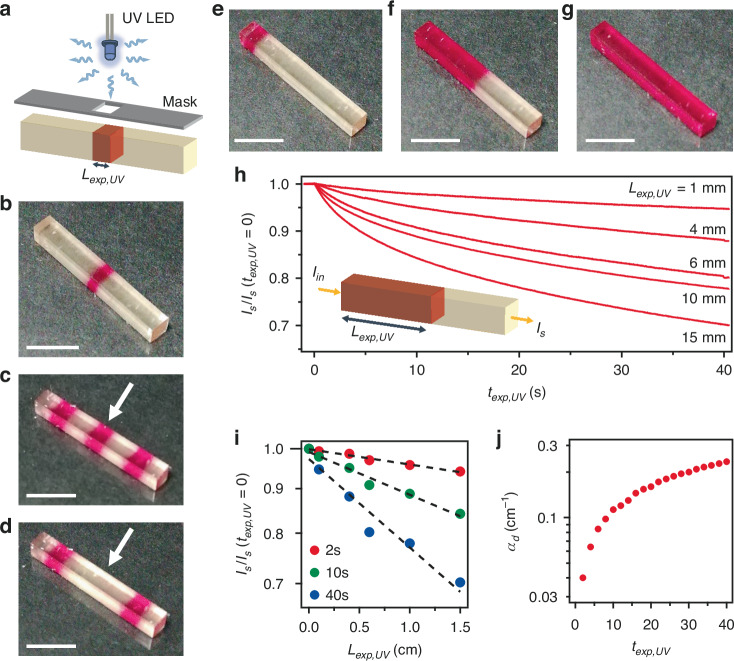
. **Spatiotemporal control of light propagation by 3D-printed photochromic objects**. **a** Scheme of the set-up used for the photoswitching through shadow masks. **b**–**g** Photographs of BTF6 printed samples, after exposure by UV light with various spatial patterns. The sample in (**d**) is obtained from the one shown in (**c**) by selectively irradiating with green light the area highlighted by the arrow. Scale bars in (**b**–**g**): 5 mm. **h** Transmitted intensity (*I*_*s*_) of a signal beam at 617 nm *vs*. UV-exposure time interval (*t*_*exp,UV*_), for samples with varied length (*L*_*exp,UV*_) of the UV-exposed region (as in **e**–**g**). From top to bottom *L*_*exp,UV*_ is: 1, 4, 6, 10, and 15 mm. Each set of data is divided by the intensity measured before UV exposure, *I*_*s*_(*t*_*exp,UV*_ = 0). Inset: schematic illustration of the propagation losses measurement. **i** Dependence of the light transmitted on *L*_*exp,UV*_, for different UV exposure times: *t*_*exp,UV*_ = 2 s (red circles), 10 s (green circles) and 40 s (blue circles). Dashed lines are fits to the data by an exponential function. **j** *α*_*d*_ coefficients at different UV exposure time intervals. Data in (**i**) and (**j**) are shown in logarithmic scale

To demonstrate the capability of encoding light propagation control with high accuracy in space and time into BTF6 printed materials, an elongated structure is produced and exposed though a shadow mask, as displayed in Fig. 3a. Figure 3b-g shows photographs of samples with various colored patterns, obtained by irradiating different areas of the 3D-printed slab. Distinct areas can be written and made colored by a suitable sequence of UV exposure (Fig. 3b,c), and then selectively erased by green light exposure (Fig. 3d). This allows us to obtain systems with precise dynamical control of light signals through the 3D-printed photochromics.

Specifically, a beam of light with intensity, *I*_*in*_, will experience an attenuation while propagating through the material: ${{I}_{s}=I}_{{in}}{e}^{-({\alpha }_{s}L+{\alpha }_{d}{L}_{\exp ,{UV}})}$, where *I*_*s*_ is the intensity transmitted by the sample, *L*_*exp,UV*_ is the length of the written photoswitched regions (see Fig. 3a, e-g, and inset of Fig. 3h), and *α*_*s*_ and *α*_*d*_ are the static and dynamic components of losses, respectively. *α*_*d*_ can be in turn controlled by the UV exposure time (*t*_*exp,UV*_) used in selective writing, mainly accounting for the additional absorption associated with photoconversion to c-BTF6.

How the intensity of a signal can be dynamically attenuated by using different *t*_*exp,UV*_ values on slabs with varied *L*_*exp,UV*_ (Fig. 3e-g) is evidenced in Fig. 3h. Here, the transmitted intensity (*I*_*s*_) is measured for a light beam (incident intensity, *I*_*in*_) passing through the printed structures, exposed to UV light for increasing times, *t*_*exp,UV*_, and for a length, *L*_*exp,UV*_ (scheme in the inset of Fig. 3h). From data shown in Fig. 3h, analyzing the dependence of the intensity of transmitted light, *I*_*s*_, on *L*_*exp,UV*_ at fixed *t*_*exp,UV*_ values (Fig. 3i) leads to determine *α*_*d*_, by an exponential fit (Fig. 3i). This study can be performed for various *t*_*exp,UV*_, and the variation of *α*_*d*_ with UV exposure time can be also precisely calibrated (Fig. 3j). Further, *L*_*exp,UV*_ and the dynamic component of losses can be both finely varied with additional green exposures, or by any proper and desired combination of UV and green light exposures, thus further enhancing the signal control both spatially and temporally. About one order of magnitude of controlled change of *α*_*d*_ is measured (Fig. 3j), an ample dynamic range that steer 3D-printed photochromic materials towards effective optical processors.

**Fig. 4:**
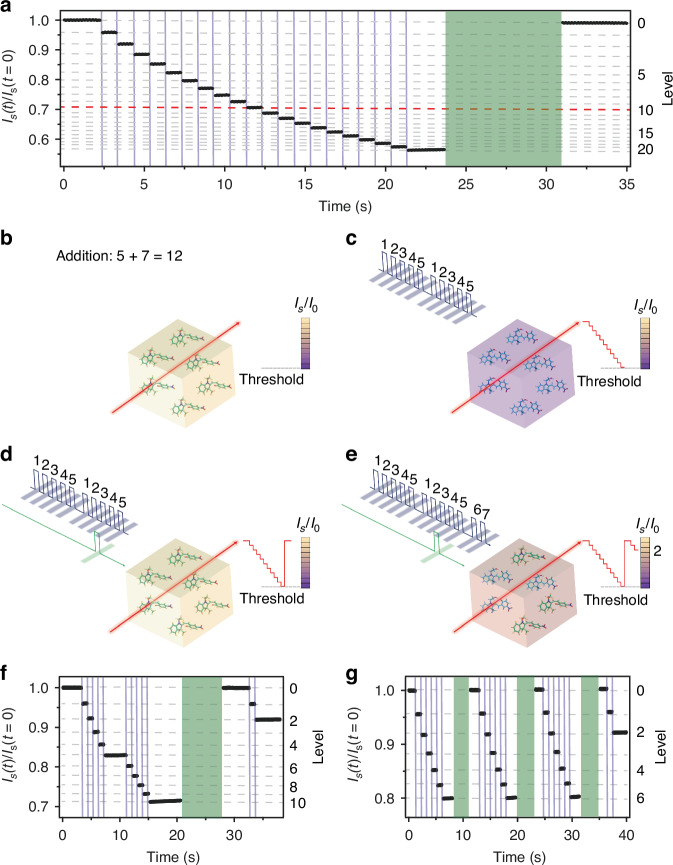
**All-optical arithmetic processing with 3D-printed photochromic devices**. **a** Intensity of probe light (617 nm) transmitted through 3D-printed SP (*χ*_SP_ = 3.5%), as measured after each UV pulse (intensity: 10 mW cm^−2^, width: 100 ms, repetition rate: 1 Hz), that gradually switches the system to the colored state. Exposure to green light (intensity: 120 mW cm^−2^) restores the initial state. The sequences of UV and green exposure intervals are shown as violet vertical lines and green shadowed areas, respectively. The red dashed line marks the probe intensity threshold, *I*_*th*_/*I*_0_ = 0.71, used for base 10 calculation (i.e., when 10 discrete levels are reached below than the initial state). **b**–**e** Scheme of the mechanism allowing for using a photochromic device for processing the sum ‘5 + 7’. Initially, the system is in its colorless form, **b**). The sum is carried out by monitoring the intensity of a probe beam, while the threshold value is known. A sequence of UV pulses corresponding to the first and second addends is sent to the device, which is gradually converted to the colored form, decreasing the intensity of the transmitted probe light, **c**). When *I*_*s*_ reaches the *I*_*th*_ value, a green pulse is sent to switch the system back to the initial, colorless state, **d**). A complete sequence of UV pulses corresponding to the addends is sent to the photochromic device, **e**). **f**, **g**) Examples of the variation of the probe signal as measured after UV/green pulses corresponding to the addition of 5 and 7, (**f**), and the division of 20 by 6, (**g**), respectively

*Calculating with light by 3D-printed photochromics*. In Fig. 4a, we show the intensity of a probe light beam transmitted by a printed photochromic device (exemplary plates in Fig. S7), monitored in real time during an exposure sequence with 20 UV pulses. Initially, the system is in a SP-rich state that is almost transparent at the wavelength of the probe beam. Each UV pulse dynamically reconfigures the photochromic device by varying the relative content of SP/MC, consequently changing the intensity of the transmitted probe light through discrete and well-defined levels. The initial state of the device can be restored by exposure to green light (Fig. 4a). Multi-step photoswitching can be also achieved at high rates (an exemplary use with a sequence of UV pulses with 10 ns duration is shown in Fig. S8). This behavior can be used for performing arithmetic operations, through approaches pioneered with phase change materials^12,13^, and here leading to fully-organic processors. A case of simple operation, such as the sum between two addends (i.e., ‘5’ and ‘7’) in base 10 is sketched in Fig. 4b-e and experimentally demonstrated in Fig. 4f. First, the chosen computation base identifies the limit level of the photochromic processor, that is a threshold level, *I*_*th*_, for the intensity of transmitted light (red dashed line in Fig. 4a).

To perform the sum of two addends, a number of UV pulses corresponding to the first addend is sent to the photochromic device, followed by a number of UV pulses corresponding to the second addend (Fig. 4c), whereas a green reset pulse is sent every time the threshold level is reached (Fig. 4c,d). The result of the sum is provided by the number of reset pulses which gives the tens, while the final level of the probe intensity gives the units (Fig. 4e). Figure 4f shows the results of such operation, ‘5 + 7 = 12’, performed by using SP/MC printed photochromics. One reset pulse is delivered after the first 10 pulses, and the final level *I*_*s*_ / *I*_0_ = 0.92 reached corresponds to the additional value of 2. Other arithmetic operations can be easily implemented by this method^12,13^. For example, the multiplication, being a series of additions, can be performed in a way similar to that shown in Fig. 4b-e. For the division, the threshold is set to the value of the divisor and a sequence of UV pulses corresponding to the value of the dividend is sent to the photochromic processor. The result is given by the number of reset pulses, with a remainder corresponding to the final level of the probe beam, as show in Fig. 4g for the operation 20 divided by 6. Calculations are also performed by printed BTF6 (Fig. S9), whose high thermal stability also allows the processor to concomitantly store its resulting configuration for long time.

The potentialities of the 3D-printed photochromics can be further developed by combining two devices, for performing the operation described above and for counting the reset pulses, respectively. To this aim, one can initialize the second photochromic processor by UV exposure to configure an initial state rich of MC molecules (colored state). Then, by sending a sequence of green pulses, the MC→SP back-conversion in the second unit is achieved through discrete levels (Fig. S10), similarly to the SP→MC conversion shown in Fig. 4a. This feature can be used for counting the number of green pulses used for resetting the first device (*A* in the set-up in Fig. S11), by sending part of their intensity (∼10%) to the second photochromic device (*B*) that has been initially colored. The result of an addition with two digits will be given by a number of unit given by *A*, whereas the number of tens will be given by the signal from *B* (Fig. S11). In this method, the photochromic devices serve as the horizontal strings of an abacus, with each green reset corresponding to a carryover operation^13^. An addition carried out by using two photochromic devices is shown in Fig. S12. Operation in other bases can also be accomplished, by shifting accordingly the threshold value (Fig. 4a).

**Fig. 5:**
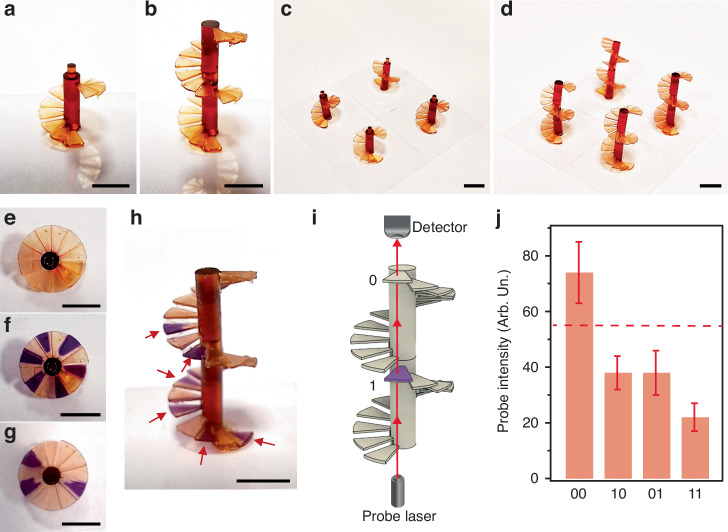
**Vertically-stacked 3D printed photochromic processors**. Photographs of a basic 3D-printed SP (*χ*_SP_ = 3.5%) device, (**a**), and of two vertically-stacked devices, (**b**), made of spiral staircases with 12 steps designed for parallel processing. Each step has a thickness of 0.46 mm. 2 × 2 array, (**c**), and vertically-stacked devices, (**d**). Photographs from the top side of the spiral-shaped devices, (**e**), and after, (**f**, **g**), selective UV photoswitching (7.6 mW cm^−2^, 20 s) of distinct steps. **h** Side-view photograph of two vertically stacked spiral-shaped components after photoswitching of selected steps (highlighted by arrows). Scale bars in (**a**–**h**): 5 mm. **i** Schematic illustration of the 3D printed devices used as multiple NOR logic gates. **j** Intensity of the probe beam measured for various UV input signals (data are average value and standard deviation measured for 10 different devices). The first (second) digit refers to the bottom (top) photochromic spiral staircase. The dashed horizontal line marks a suitable threshold value

We point out that 3D geometries can lead optical processors to new architectures and volume combination capabilities. For instance, multiple photochromic systems can be integrated by using 3D spiral-shaped components for parallel processing, as shown in Fig. 5. Here the system embeds 12 photochromic processors with SP/MC (each device being a step of the spiral staircase), while the spiral-shaped components can be stacked vertically (Fig. 5b) for those operations that require the use of multiple photochromic elements placed in series, and arranged in arrays (Fig. 5c,d) for parallel processing. Each photochromic element can be then individually addressed (Fig. 5e-h) to obtain multiple logic gates. Various schemes can be adopted to this aim. For instance, input UV light can be used to straightforwardly obtain a NOT gate^19^ by SP printed processors. The gate is colorless when the input UV light is off (“0” input), hence the intensity of a probe beam with wavelength within the absorption band of the MC will be transmitted, thus effectively serving as “1” output. By switching the UV light on (“1” input”) the photochromic logic gate will be switched to the colored state leading a probe beam intensity to high attenuation due to the MC absorption (“0” output). Each spiral-shaped structure, as those photographed in Fig. 5, thus supports a NOT gate at each step element, while placing two photochromic spirals in series (Fig. 5i) leads to 12 NOR gate^19^ by choosing a suitable threshold value for the probe intensity (Fig. 5j, S13 and Table S1).

## Discussion

3D printed photochromic materials are demonstrated with a SP and a diarylethene derivative, featuring absorption and fluorescence controllable by optical stimuli. By localizing exposure regions in the 3D printed structures and by controllably varying exposure intervals, the propagation of probe signals through the materials might be efficiently controlled, thus leading to a new and versatile class of molecular optical processors and logic operators. These printable photochromic materials feature high versatility in terms of achievable color conversion, since the sample composition can be varied in an ample range to obtain different optical transmission properties in the printed structures (Fig. S14). The architecture of so-obtained components can be specifically designed, and exposure sequences varied in intensity and duration, to induce well-defined, precisely calibrated changes of the device response. Interestingly, for computational applications the energy for achieving the probe beam intensity variation corresponding to the threshold value is in the range 10–30 mJ cm^−2^, that is about one order of magnitude lower than that required to perform similar operations with other materials.^12^

Additive manufacturing might be further used to enhance properties of active molecular dopants, expanding their calculating and memory capabilities and their potential for parallelization in two and three dimensions. Such features are relevant for the automated control and routing of the probe and excitation light beams used in the operations. These results pave the way for all-organic all-optical computing platforms which can be integrated in 3D architectures, and in new schemes for advanced microscopy, sensing, and physical intelligence.

## Materials and methods

### Materials

A pre-polymer mixture made of the oligomer BEDMA (molecular weight, *M*_*n*_ ~ 1700 g mol^−1^, Sigma-Aldrich, Darmstadt, Germany) and the TPO photoinitiator (IGM Resins, Waalwijk, The Netherlands) is used with weight/weight ratio (χ_TPO_) between the photoinitiator and the BEDMA oligomer varying in the interval 0.1–1%. The dissolution of the TPO in BEDMA is achieved by alternating the use of a vortex and an ultrasonic bath at 40 °C. The printable photochromic materials are made by adding either the SP (Sigma-Aldrich) or the BTF6 (Kanto Chemical, Tokyo, Japan) to the TPO/BEDMA mixtures. The dissolution of the photochromic molecules in the mixtures is achieved by using a vortex and an ultrasonic bath at 40 °C. To investigate how photochromic molecules dissolve in the pre-polymer, various solutions are made by using χ_TPO_ = 0.1% and χ_TPO_ = 1% and by increasingly adding the amount of photochromic molecules in steps of χ_SP/BTF6_ = 0.25%, following the same preparation procedure described above. High amounts of photochromic molecules are preferred since allowing the absorption of the probe light by the printed structures to be enhanced, and larger variations of the transmitted intensity of the probe light beams (617 and 638 nm) to be obtained. Afterwards, 100 µL of each solution are poured on a coverslip and inspected by optical and fluorescence microscopy (Figs. S15, S16) by using an upright stereomicroscope (MZ16 FA, Leica, Wetzlar, Germania). By such analysis, χ_SP_ = 3.5% and χ_BTF6_ = 3% are found to be the highest weight ratios that allow homogeneous solutions to be obtained. In fact, by further increasing the amount of photochromic molecules in the pre-polymer, undissolved particles are found in the mixtures (see Figs. S15b,d and S16b,d). Moreover, varying χ_TPO_ in the interval 0.1%-1% by keeping χ_SP_ = 3.5% and χ_BTF6_ = 3%, 3D printing exposure times are optimized, as described below. Samples made with lower contents of TPO require longer printing times. χ_TPO_ = 0.5% is selected as the value allowing each layer of the 3D objects to be printed in 6–12 s, keeping photoswitching fully effective.

### 3D printing

The samples are printed by DLP using the Micro Plus HD (ENVISIONTEC, Gladbeck, Germany) system, equipped with a light-emitting diode (LED) light source for photopolymerization (peak wavelength: 405 nm). The photopolymerization time of the layers is optimized for each pre-polymer mixture (12 and 6 s, respectively), by the method reported in the SI (Fig. S17). The first three layers are printed with higher photopolymerization times (2 seconds more than the other layers) in order to let the printed object adhere to the print holder. After printing, the samples are rinsed in isopropanol, dried under a nitrogen flow and inspected by using an upright stereomicroscope. Before optical characterization and use, the samples are exposed to green light by using a 520 nm LED source (H-G3, Ultrafire; intensity: 30 mW cm^−2^; duration: 30 s). Samples with thicknesses varying from a few hundreds of micrometers (Fig. S7), up to mm (Figs. 1c–f, and 3b-g) show similar color conversion properties.

### Optical characterization

The optical transmittance spectra of printed structures (size: 10 × 10 × 0.18 mm^3^) are measured by a spectrophotometer (Lambda 950, Perkin Elmer, Waltham, Massachusetts, USA). The colored states are achieved by 30 s exposures to 365 nm light (EN-180L/FE, Spectroline, Melville, NY, USA; intensity: 0.7 mW cm^−2^). The back-conversion is performed by 30 s of exposure to the 520 nm LED source. Back-conversion in dark conditions is assessed by measuring the transmission spectra after illumination by UV light (30 s, 405 nm LED source). The PL spectra of the 3D printed photochromic samples after UV light exposure is excited by a 532 nm diode-pumped solid-state laser (DJ532-40, Thorlabs, Newton, New Jersey, USA). The light emitted by the samples is collected by a lens and coupled to an optical fiber, connected to a spectrometer (Flame, Ocean Optics, Orlando, FL, USA). Real time measurements of the variation of the transmittance spectra of printed samples (Figs. S4 and S5) are carried out by using a LED emitting at 365 nm (M365LP1, Thorlabs, intensity: 3 mW cm^-2^) as UV light source and the 532 nm laser as the green one (intensity: 13 mW cm^−2^ for SP and 6.6 mW cm^−2^ for BTF6). For probing the variation of the spectra of the light transmitted by the samples, the output beam of a broadband light source from a fiber-coupled deuterium-halogen lamp (DH-2000-BAL, Ocean Optics) is sent onto the UV/green irradiated area of samples while the transmitted light is collected by a lens and sent to a fiber-coupled spectrometer (Flame, Ocean Optics) for spectral analysis. The spectra of the transmitted light are measured while the samples are illuminated with either UV or green light, and normalized by data collected by the same experimental set-up without the sample. Attenuation coefficients are measured by using the UV LED at 365 nm for switching BTF6 from the open to the closed form. Rectangular shadow masks with different apertures are used to selectively photoisomerize specific areas of the sample, while a mechanical shutter allows for controlled exposure time. The light emitted by a 617 nm LED (LED610L, Thorlabs) is used as a probe beam in a transmission mode (Fig. S6). In particular, the 617 nm light is sent through the printed objects and the sample-transmitted intensity is measured by a Si photodiode (DET100A, Thorlabs).

### Photoswitching cycles and computing

The set-up for photoswitching cycling and optical arithmetic processing experiments are schematized in Figs. S6 and S11, respectively. UV pulses are generated by modulating the driving current of a 375 nm laser by a pulse generator, whereas green pulses are generated by a mechanical shutter modulating light from 561 nm laser (L6Cc, OXXIUS, Lannion, France). To probe samples in real time, experiments in transmission mode are performed, by using light from the 617 nm LED, and measuring intensity by DET100A Si photodiodes. A 638 nm laser beam (L6Cc, OXXIUS) can be also used as a probe beam (Fig. S11). The nanosecond switching of the 3D printed photochromic devices is also performed by using the third harmonic (355 nm) of a Nd:YAG laser (10 ns pulse duration: 10 ns, repetition rate: 10 Hz), while the 617 nm LED is used as the probe light.

## Supplementary information

Supplementary Information

## Data Availability

The data that support the findings of this study are available from the corresponding authors upon reasonable request.
