# Supplementary material for: All-optical processors by 3D printable photochromic materials
Source: Light Sci Appl. 2025 Oct 22;14:375. doi: 10.1038/s41377-025-01974-z (PMC12546934; doi:10.1038/s41377-025-01974-z)
Supplement: Supplementary file 1 — Supplementary Information [file 41377_2025_1974_MOESM1_ESM.pdf]

Supplementary Information

All-optical processors by 3D printable photochromic materials

Francesca D'Elia[1], Lorenzo Lavista[2,3], Sibilla Orsini[3], Andrea Camposeo[3,*]

Dario Pisignano[2,3,*]

[1]NEST, Scuola Normale Superiore, Piazza S. Silvestro 12, I-56127 Pisa, Italy

[2]Dipartimento di Fisica, Università di Pisa, Largo B. Pontecorvo 3, I-56127 Pisa, Italy

**E-mail: dario.pisignano@unipi.it*

[3]NEST, Istituto Nanoscienze-CNR and Scuola Normale Superiore, Piazza S. Silvestro 12, I-56127 Pisa, Italy

**E-mail: andrea.camposeo@cnr.it*

S1. Absorption, photoswitching, and photoluminescence properties

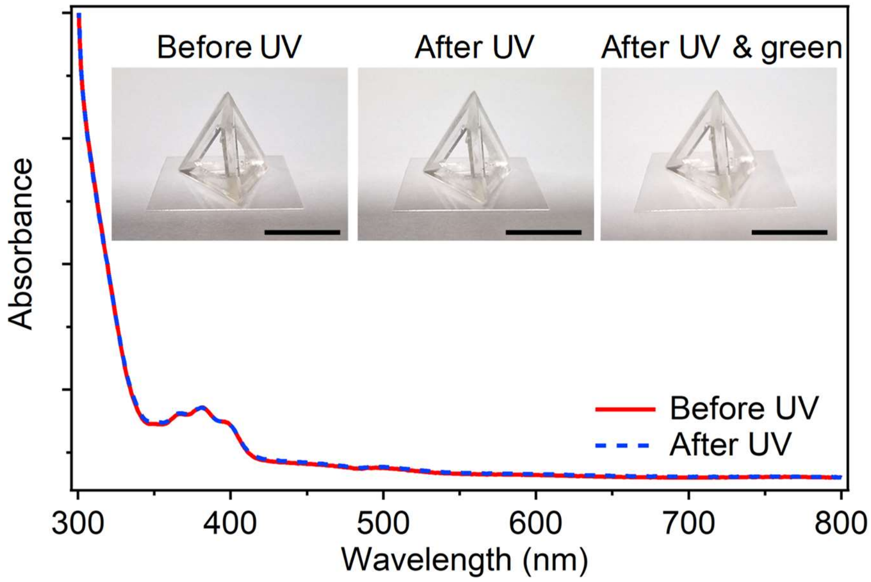

Figure S1. Absorption spectrum of BEDMA, before and after irradiation with UV light (wavelength: 365 nm; intensity: 3 mW cm^{-2}; exposure time: 120 s). Sample size 10×10×0.18 mm^3. The insets show photographs of a 3D printed pyramid made of BEDMA, before UV irradiation (left), after UV irradiation (middle), and after an additional green irradiation (right). Scale bars: 10 mm. The UV and green irradiation conditions: 30 s exposure to 365 nm light with 0.7 mW cm^{-2} intensity, 30 s of exposure to 520 nm light with 30 mW cm^{-2}, respectively.

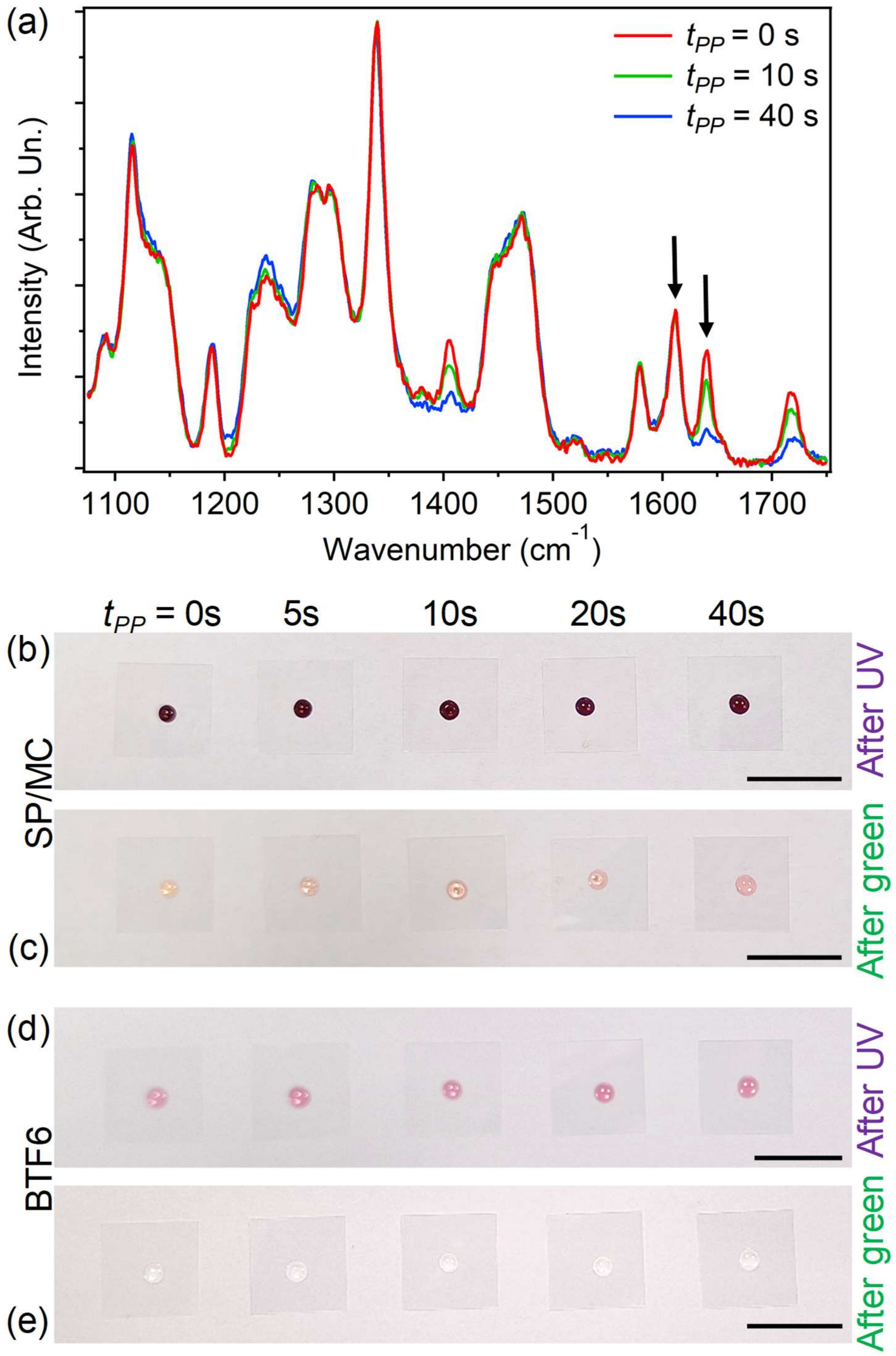

Figure S2. a) Raman spectra of TPO/BEDMA samples with SP. Samples are either not photopolymerized (red curve), or photopolymerized by 405 nm light (intensity: 6 mW cm^{-2}) for different times, t_{PP} = 10 s and 40 s (green and blue curves, respectively). While the intensity of the peak at 1612 cm^{-1} does not vary with the UV exposure, the peak at 1640 cm^{-1} is strongly weakened due to the increased crosslinking of the BEDMA matrix [the two Raman modes are

highlighted by vertical arrows in panel (a)].[S1, S2] The degree of crosslinking, C_R, for the photochromic compound can be determined by using the expression:[S1,S2] C_R=1-[(I_{1640}/I_{1612})/(I_{1640}/I_{1612})$_{ref}$], where I_{1640} and I_{1612} are the intensities of the Raman modes at 1640 cm^{-1} and 1612 cm^{-1}, respectively, and the ratio (I_{1640}/I_{1612})$_{ref}$ is measured at t_{PP}=0. C_R of about 30% and 80% are found for t_{PP} = 10 and 40 s, respectively. b)-e) Photographs of TPO/BEDMA samples doped with SP (b,c) and BTF6 (d,e) in their colored (b,d) and colorless (c,e) forms. The samples are obtained by depositing 5 µL droplets of pre-polymer on a glass substrate. The pre-polymer droplets are photopolymerized by exposure with 405 nm light (intensity: 6 mW cm^{-2}) for different times, t_{PP} = 0, 5, 10, 20 and 40 s. After photopolymerization, the samples are exposed to UV light (wavelength: 365 nm, intensity: 0.6 mW cm^{-2}) for 30 s to induce SP→MC (b) and o-BTF6→c-BTF6 (d) conversion, and then to green light (wavelength: 520 nm, intensity: 47 mW cm^{-2}) for 60 s to induce the MC→SP (c) and c-BTF6→o-BTF6 (e) back-conversion. Scale bars: 2 cm.

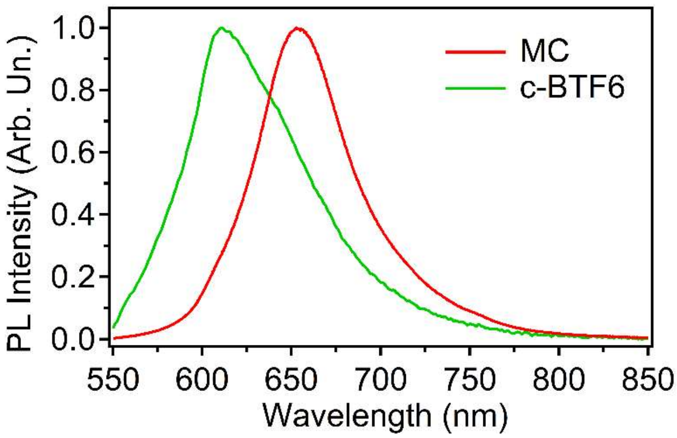

Figure S3. Photoluminescence (PL) spectra of merocyanine (MC, red line) and of the closed form of the diarylethene derivative (c-BTF6, green line) in TPO/BEDMA printed samples. The samples are exposed with UV light before measuring the PL. The PL spectra are measured by exciting the samples with a 532 nm laser and collecting the emission through a 550 nm longpass filter.

S2. Real-time monitoring of photoswitching

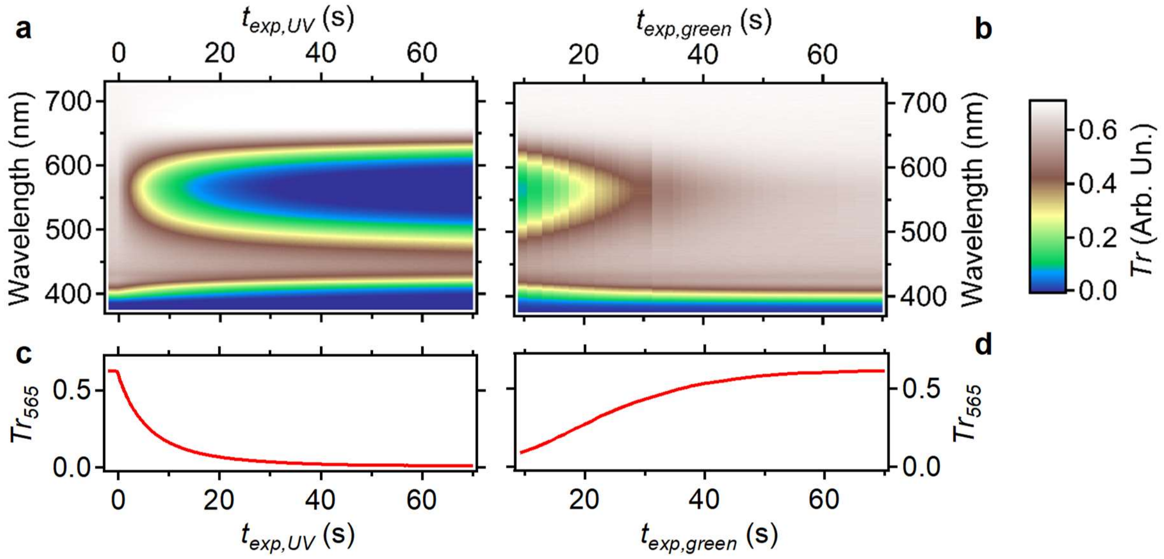

Figure S4. a,b) Transmission spectra of TPO/BEDMA with SP/MC following different UV a) and green b) light exposure times ($t_{exp,UV}$ and $t_{exp,green}$, respectively). c,d) Corresponding temporal variation of the transmission at 565 nm (Tr_{565}).

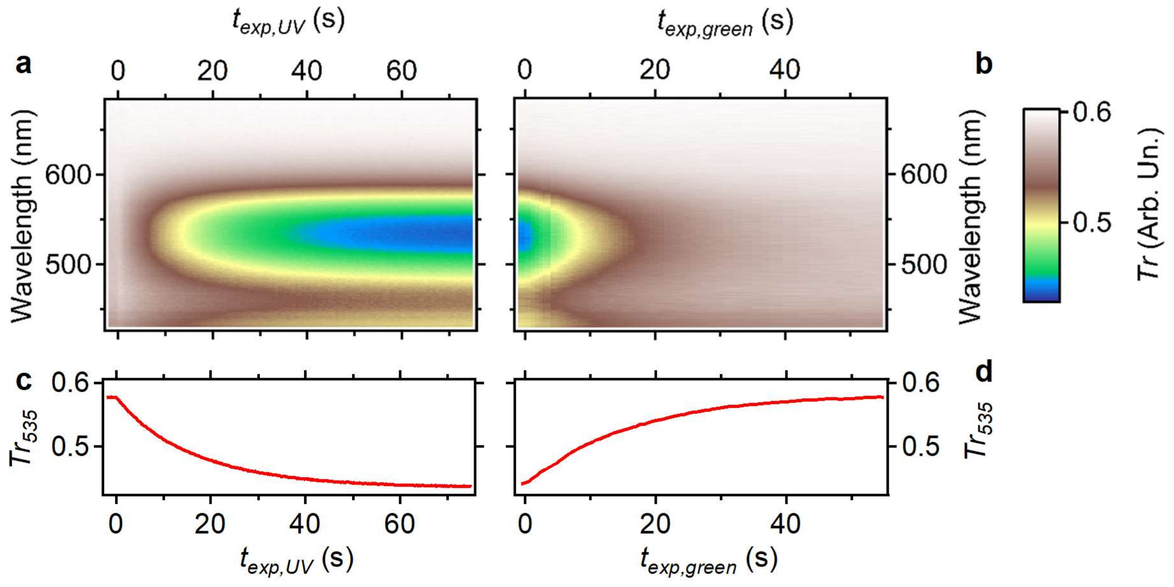

Figure S5. a,b) Transmission spectra of TPO/BEDMA with BTF6 following different UV a) and green b) light exposure times. c,d) Corresponding temporal variation of the transmission at 535 nm (Tr_{535}).

S3. Photoswitching cycles and thermal back-conversion

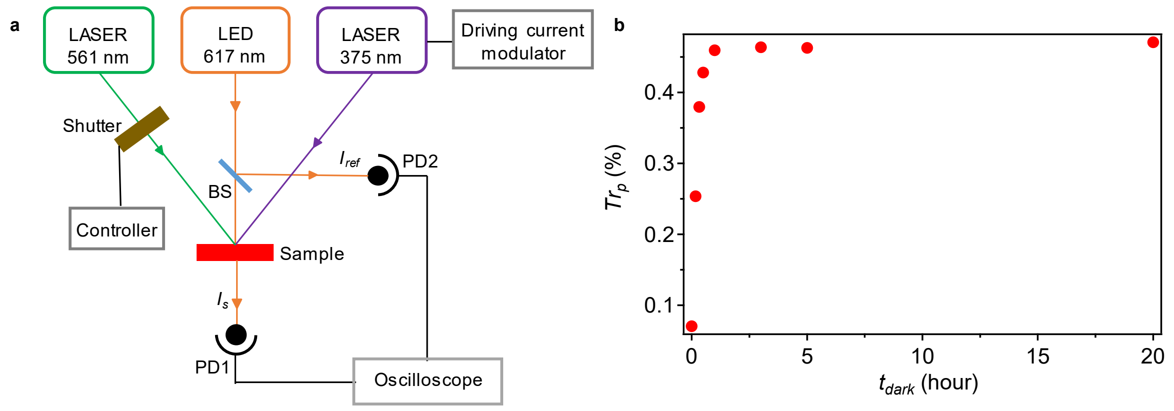

Figure S6. a) Schematic illustration of the set-up used for characterizing the fatigue properties of the photochromic printed samples. BS: beam splitter; PD1,PD2: photodiodes. The signals $I_T(t)$ shown in Figure 2a,b are obtained as: $I_T(t) = \frac{\frac{I_S(t)}{I_{ref}(t)}}{\frac{I_S(t=0)}{I_{ref}(t=0)}}$, where $I_s(t)$ is the intensity of the probe beam transmitted by the sample and measured by PD1, and $I_{ref}(t)$ is the reference intensity measured by PD2. b) Transmittance of the SP/MC printed samples (at 568 nm) *vs*. storage time in dark (t_{dark}). Some transmission spectra used for the analysis are shown in Figure 2e of the main manuscript.

S4. Calculating with light by 3D-printed photochromics

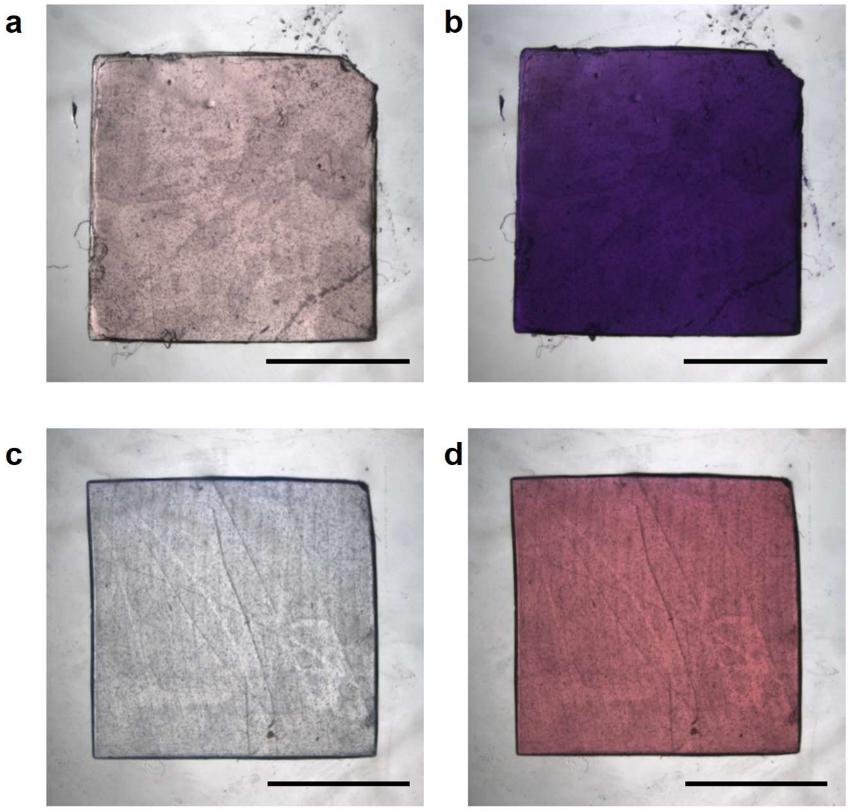

Figure S7. a-d) Photographs of simple 3D printed SP (a,b) and BTF6 (c,d) devices used for all-optical arithmetic processing. The photographs show the devices before (a,c) and after (b,d) 365 nm UV light exposure. Scale bars: 5 mm.

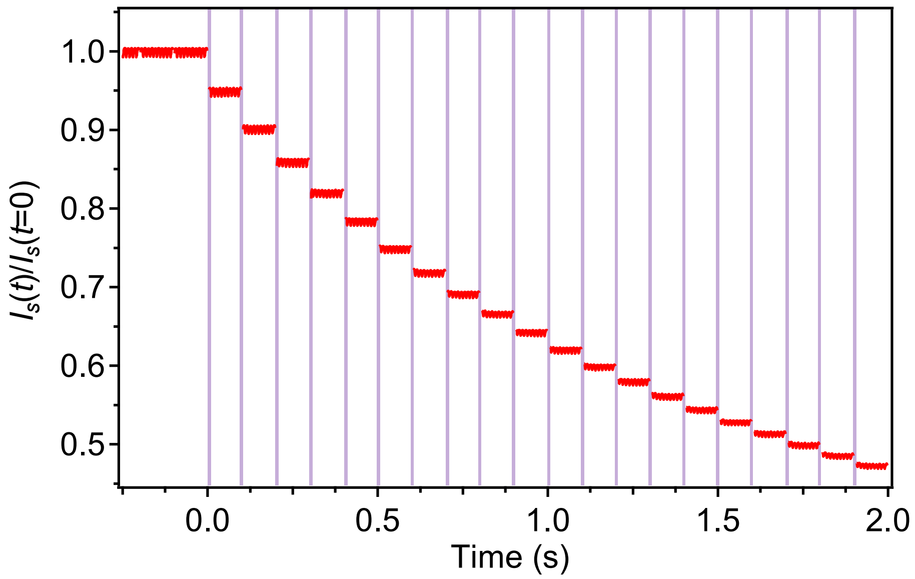

Figure S8. Intensity (I_s) of a probe light beam at 617 nm (red data), transmitted through 3D-printed SP (χ_{SP} = 3.5%) for a sequence of UV pulses (Nd:YAG laser source, wavelength: 355 nm, fluence: 3 mJ cm^{-2}, width:10 ns, repetition rate: 10 Hz). The UV exposure intervals are shown as violet vertical lines.

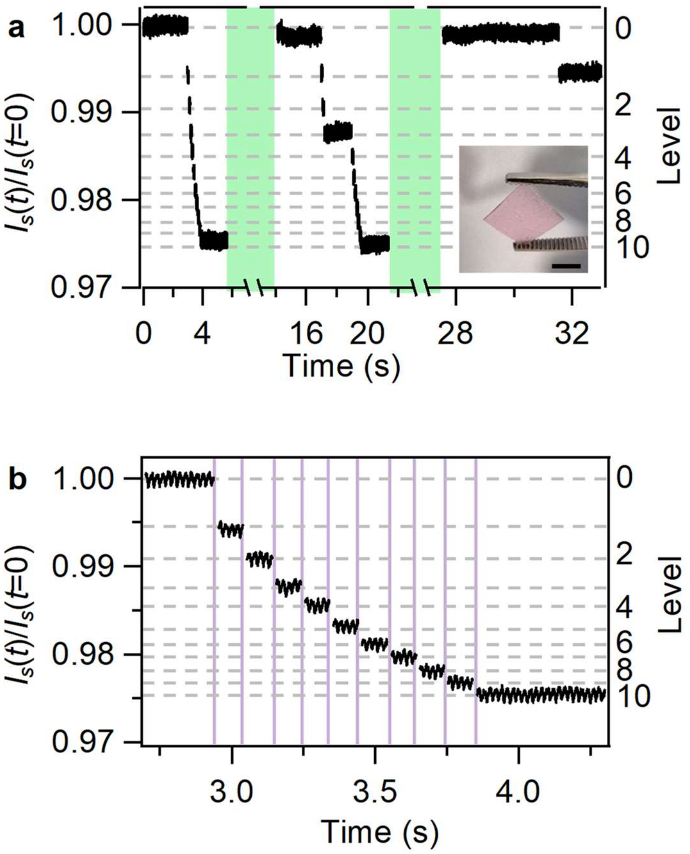

Figure S9. a) Intensity (I_s) of the probe light (617 nm), transmitted through 3D-printed BTF6 χ_{BTF6} = 3%), as measured during UV pulse sequences (intensity: 95 mW cm^{-2}, duration: 10 ms, repetition rate: 1 Hz) corresponding to the addition of '13' and '8'. Exposure to green light (intensity: 50 mW cm^{-2}, green shadowed area) restores the initial state. The dashed horizontal lines are guide for the eyes. Inset: photograph of a 3D-printed BTF6 device. Scale bar: 5 mm. b) Zoomed view of the behavior of the probe signal during the first 10 UV pulses. The violet vertical lines highlight the intervals in which the UV pulses are on.

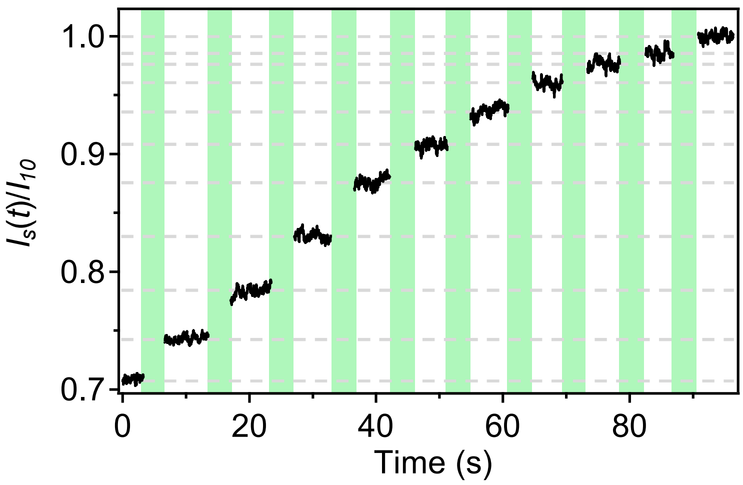

Figure S10. Intensity (I_s) of the probe light at 638 nm (black data), transmitted through 3D-printed SP/MC (χ_{SP} = 3.5) during a sequence of UV/green pulses. I_{10} is the average intensity of the probe beam after the 10th interval of green exposure. The sample is first converted to the colored state by UV (365 nm, intensity: intensity: 7.6 mW cm^{-2}, duration 30 s). The sequences of green exposure intervals (intensity: 2 mW cm^{-2}) are shown as green shadowed areas.

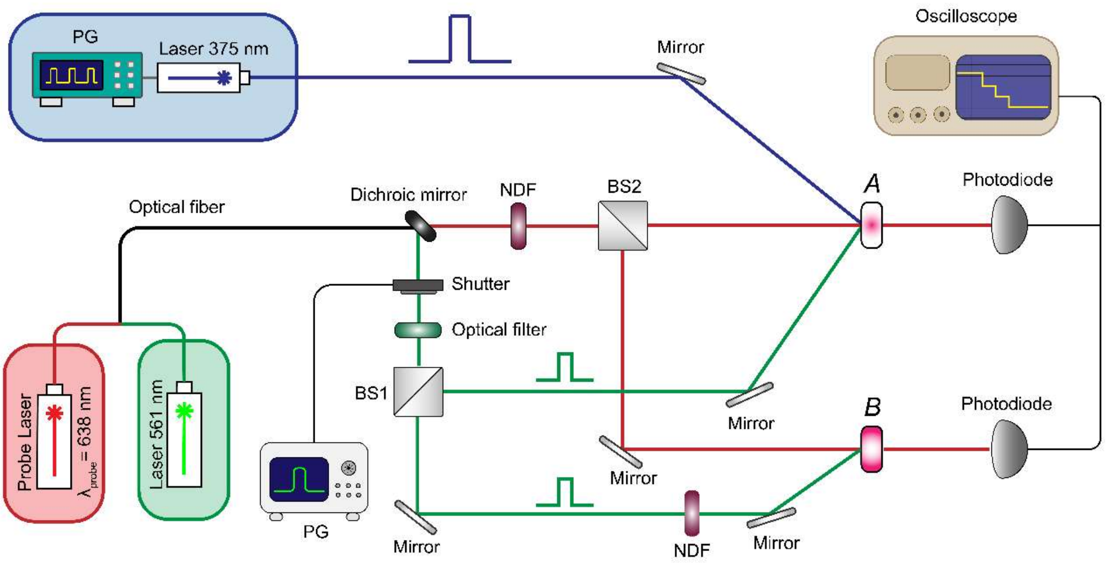

Figure S11. Scheme of the set-up used for arithmetic processing with two printed photochromics. BS1: 10/90 beam splitter; BS2: 50/50 beam splitter; PG: pulse generator; NDF: neutral density filter.

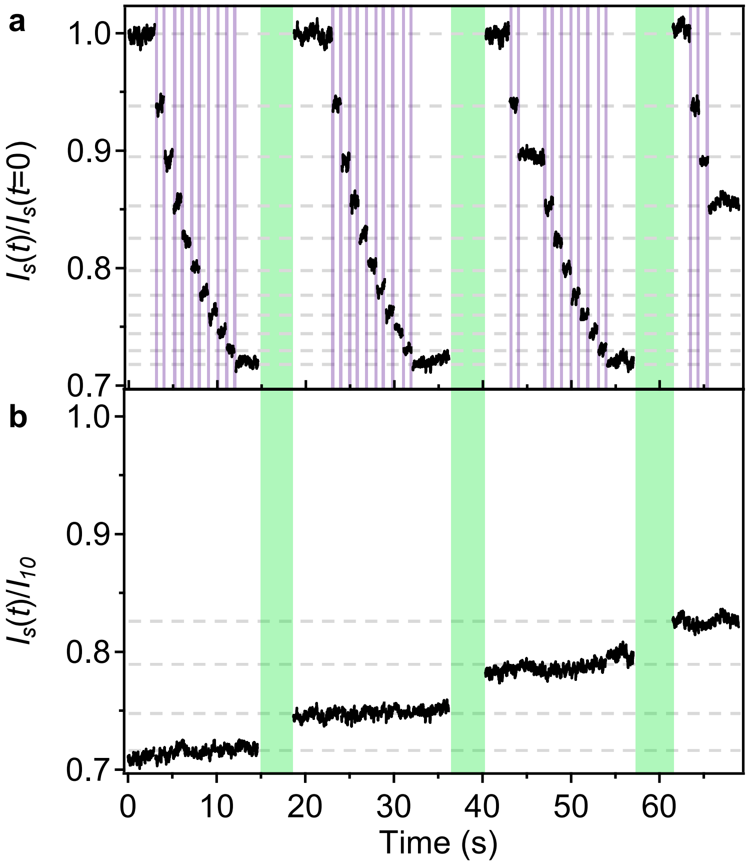

Figure S12. a) Intensity (I_s) of the probe light at 638 nm (black data), transmitted through *A* (a) and *B* (b) 3D-printed SP/MC, for a sequence of UV/green pulses corresponding to the addition of '22' and '11' (*A* and *B* as in Fig. S9). Each UV pulse has intensity of 10 mW cm^{-2} and width of 100 ms. The intensity of green light sent to *A* (*B*) is 120 mW cm^{-2} (2 mW cm^{-2}). *B* is first converted to the colored state by UV irradiation (365 nm, intensity: intensity: 7.6 mW cm^{-2}, duration 30 s). The sequences of UV and green exposure intervals are shown as violet lines and green shadowed areas, respectively.

UV_{bottom}	UV_{top}	I_{probe} (Arb. Un.)	**Threshold 55 (Arb. Un.)** ⇨	UV_{bottom}	UV_{top}	I_{probe} (Arb. Un.)
Off	Off	74±11		0	0	1
On	Off	38±6		1	0	0
Off	On	38±8		0	1	0
On	On	22±5		1	1	0

Table S1. Left: Dependence of the intensity, I_{probe}, of the probe beam (638 nm), passing through two spiral-shaped 3D-printed photochromic processors, on the UV irradiation of a single step of the bottom and top staircase (Figure 5i). By selecting a threshold value of 55 (arb. un.) for the probe intensity, the truth table of a NOR gate is obtained (right).

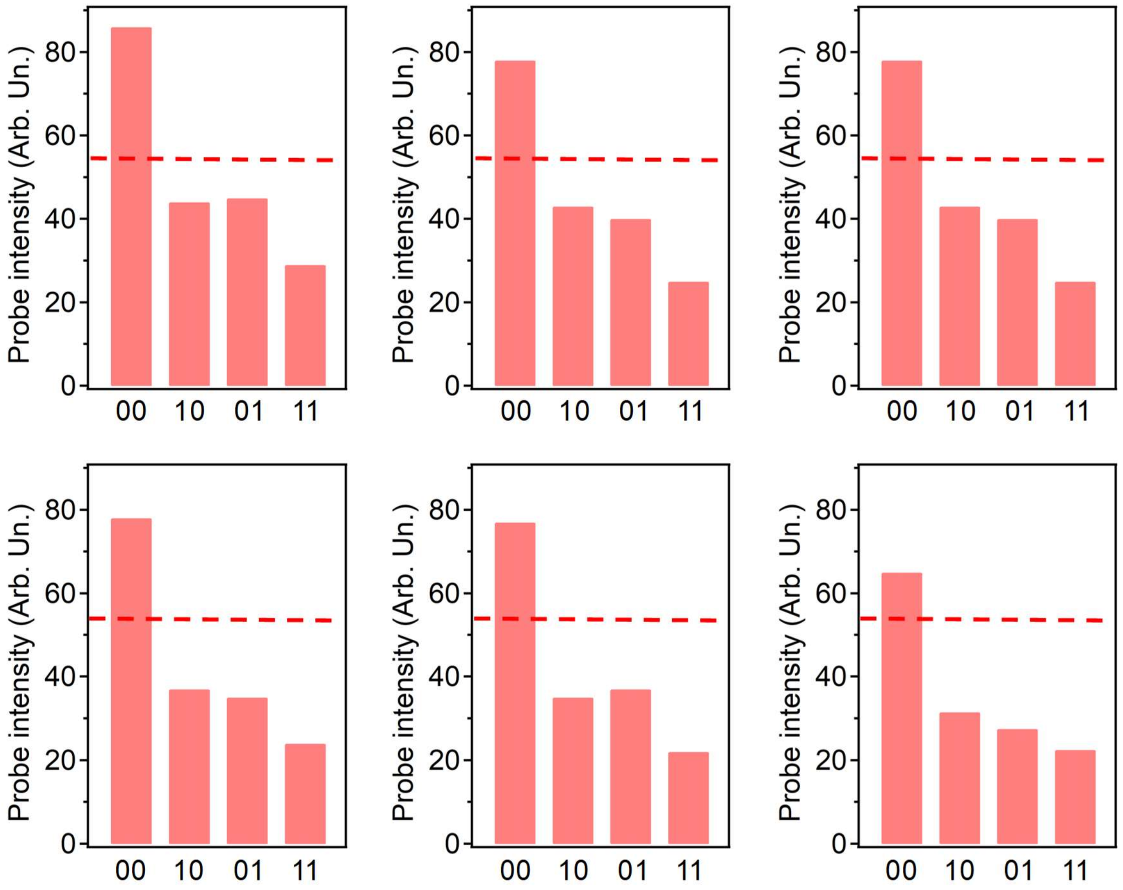

Figure S13. Intensity of the probe beam measured for various UV input signals (data are measured in different positions of the device shown in Figure 5i). The first (second) digit refers to the bottom (top) photochromic spiral staircase. The dashed horizontal line highlights the threshold value (Table S1).

S5. 3D printing of photochromic objects by DLP

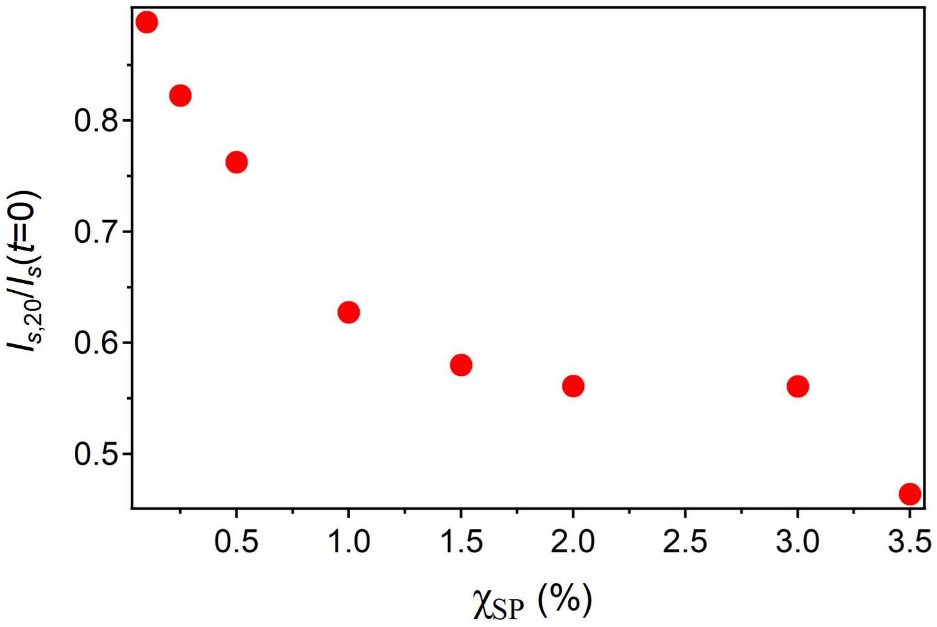

Figure S14. Intensity of probe light (638 nm) transmitted through 3D-printed BEDMA with SP, as measured after 20 UV optical pulses (wavelength: 375 nm, width: 100 ms, repetition rate: 1 Hz) as a function of the weight ratio, χ_{SP} (χ_{TPO}=0.5%).

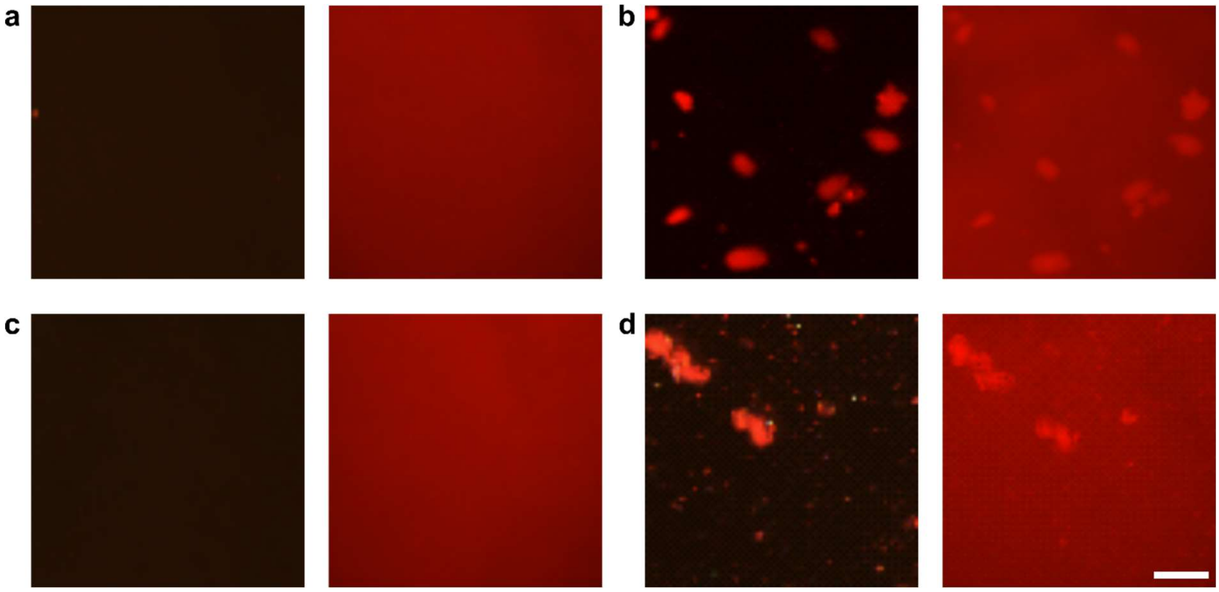

Figure S15. a-d) Dark field micrographs (left images) and fluorescence micrographs (right images) of BEDMA pre-polymers with different content of TPO and SP: a) χ_{TPO} = 0.1%, χ_{SP} = 3.5%; b) χ_{TPO} = 0.1%, χ_{SP} = 3.75%; c) χ_{TPO} =1%, χ_{SP} = 3.5%; d) χ_{TPO} = 1%, χ_{SP} = 3.75%. Scale bar: 500 μm.

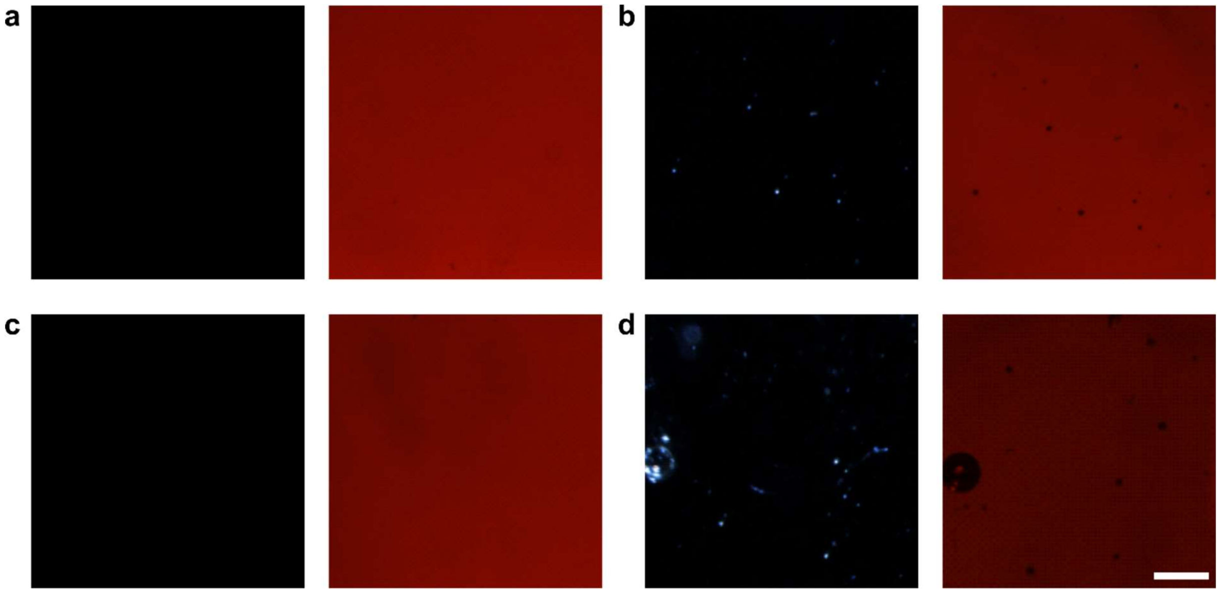

Figure S16. a-d) Dark field micrographs (left images) and fluorescence micrographs (right images) of BEDMA pre-polymers with different content of TPO and BTF6: a) χ_{TPO} = 0.1%, χ_{BTF6} = 3%; b) χ_{TPO} = 0.1%, χ_{BTF6} = 3.25%; c) χ_{TPO} =1%, χ_{BTF6} = 3%; d) χ_{TPO} = 1%, χ_{SP} = 3.25%. Scale bar: 500 μm.

S5.1 Variation of 3D printing exposure time

In order to determine optimal DLP printing processes for the TPO/BEDMA pre-polymers doped with either SP or BTF6, squared slabs with size 10×10×0.18 mm^3 are printed with varied UV photopolymerization time. After the printing process, the samples are rinsed in isopropanol, dried under a nitrogen flow, and inspected by an upright stereomicroscope (Figure S13a,b). A morphological analysis is performed by measuring the top area of each 3D-printed structure (A_M), comparing it to the design value, A_D (i.e. 1 cm^2), and calculating the normalized difference: $\Delta A = |(A_D - A_M)/A_D|$. The results are shown in Figure S13c,d. For low photopolymerization times the printed structures are not fully polymerized, while the areas of samples printed by too high photopolymerization times are larger than the design value, due to overexposure. Optimal printing conditions are found for 11.5-12.5 s for SP and 5.5-6.5 s for BTF6.

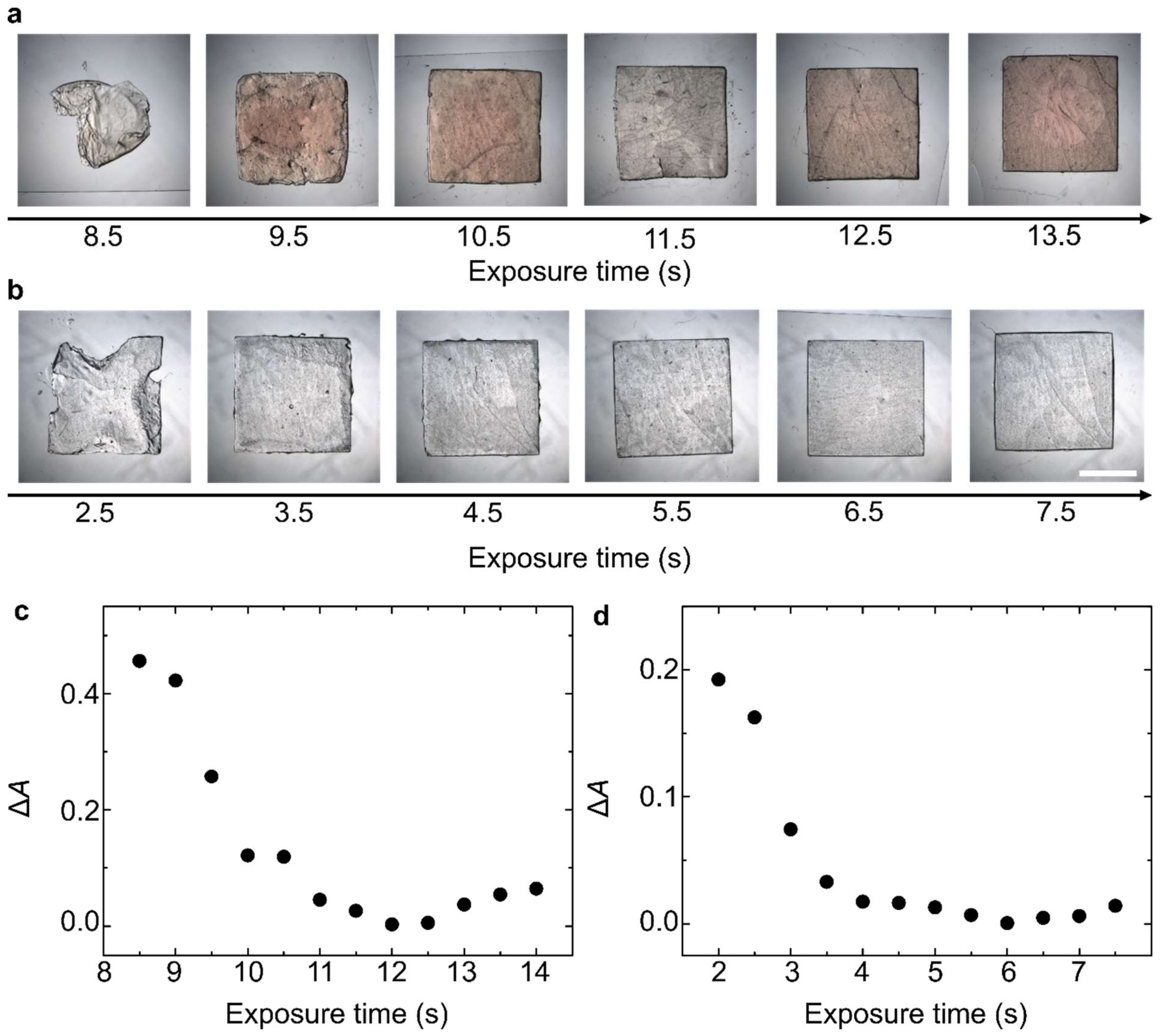

Figure S17. a,b) Photographs of 3D printed squared samples (design: $10\times10\times0.18$ mm^3) made by DLP at varied exposure times, with pre-polymers with χ_{TPO} = 0.5 % and either χ_{SP} = 3.5 %, a), or χ_{BTF6} = 3%, b). Scale bar: 5 mm. c,d) ΔA vs. exposure time of printing processes for samples with SP, c), and BTF6, d).

References

[S1] Y. Cai, J. L. P. Jessop, *Polymer* **47**, 6560 (2006).

[S2] M. Par, N. Spanovic, T. T. Tauböck, T. Attin, Z. Tarle, *Scientific Reports* **9**, 17245 (2019).
